# Unsound Conduct and Unsound Mind

**Published:** 1918-09-28

**Authors:** 


### PAPERS ON MADNESS.

### VI. Unsound Conduct and Unsound Mind.

An opinion is very widely prevalent among medical men that a person cannot be certified as mad unless he or she suffers from some delusion, or unless he or she is dangerous to others or suicidal. These are total and utter mistakes, that frequently lead to disaster. There is not the slightest foundation for them: yet they are so widely prevalent that they are acted upon by many medical men, that barristers occasionally formulate them in courts of law, and that even alienists engaged in the care of the insane, men who most certainly ought to know better, sometimes express them in their writings, though it is to be hoped they do not act upon them. The actual law upon the subject, as it really is, cannot be much better expressed than it is expressed in the terms of the Urgency Certificate upon which a patient may be removed to an asylum. The Act says that it is lawful to detain a patient under care and treatment if it is expedient *for the welfare of the patient* or for the public safety. It is particularly to be noticed that in the consideration of the Legislature the welfare of the patient comes first. This is the first thing to be considered. This is what is to carry most weight in the mind of the certifier. He is to consider whether it is to the advantage of the patient himself to be detained under *care and treatment*, and if it appears to be expedient—not necessary, but expedient—for the welfare of the patient, the certificate ought to be made. This is the official requirement of the Act of Parliament. This is the requirement of the Legislature. It says nothing about delusion, and it says nothing in the requirement about violence. All that the Legislature requires in order to legalise the detention of a patient is that it should be expedient for his own welfare that he should be detained. Observe, it does not say "expedient for his safety." No doubt his safety is included in his welfare, but his welfare includes very much more than his safety. It includes, in the first place, his chance of recovery. If it appears likely that he would recover sooner by being placed under detention than by being left at large, then it is lawful to certify him and place him in detention, whether he has a delusion or not, and whether he is violent or not, and whether he is suicidal or not. If his conduct is such—remember, it is conduct alone that matters—if his conduct is such as to imperil his reputation, then it is expedient for his welfare that he should be placed under care, and then he may lawfully be certified as insane. It may be that his conduct is indecent, lascivious, and calculated to make him an object of attention by the police, and then of course it is expedient for his welfare that he should be certified and placed under restraint; but again, it is not in the least necessary that his conduct should be of this description. If he is squandering his money or his property recklessly, or indulging in reckless speculation, and there is reason to believe that this conduct is mad, then it is expedient for his welfare that he should be certified and detained, and this may lawfully be done. But even as great an aberration of conduct as this is not necessary. If the normally quiet, reticent, self-restrained man becomes very talkative and boisterous and expansive, so as to diminish his credit and reputation with his patients if he is a doctor, or with his clients if he is a lawyer, or with his customers if he is a man of business, or with his employers, whatever his capacity, it is expedient for his welfare that he should be certified and placed under care and treatment, as "a person of unsound mind" so called; really, of course, as a person of unsound conduct. In all the respects that have been enumerated, what is considered in forming a decision whether to certify him or not is not his mind, but his conduct; not what he feels or thinks, but what he says and does; not whether he has a delusion or not, but whether he does or does not act as a sane man. That is the test, and that is the criterion, and whether or not his mind is disordered never enters into the consideration of the certifier in the least.

Of course, if we find in our investigation of him evidence of delusion, there is no reason why we should not add this evidence to our certificate as a make-weight. It will help to corroborate our diagnosis, for when a man is mad his mind is disordered, and if we find evidence of certain kinds of disorder in his mind, that is corroborative evidence that he is mad; but it should be used only as corroboration. It is not enough by itself. However gross his delusions may appear to be, we are not justified in certifying him as mad unless his conduct is in some respect that of a madman. Of course, in practice, we do not find gross delusion without some disorder of conduct; but the practice of depending on delusion alone has given rise to most deplorable mistakes, and will, no doubt, give rise to more, especially as sufficient care is seldom taken to make sure that what appears to be a delusion really is a delusion. A patient has been certified as insane on the ground that he had a delusion that his wife was unfaithful to him, that he was ruined, that the police would be after him for some offence, that his partner was robbing him, and so forth, "delusions" that turned out ultimately to be solid facts, only too well substantiated by subsequent events. These are terrible mistakes, but they have been made, and they will be made again if doctors persist in the mistaken practice of depending on delusion as the only proof of madness, or even as by itself conclusive proof of madness. Whether Swedenborg was mad cannot now be ascertained, but it is certain that his followers would indignantly repudiate that he was; yet Swedenborg undoubtedly suffered from delusions.

Of course, if a man's conduct is such as to imperil either his own safety or that of other people, he can be certified as mad, and even those doctors

☞ Previous articles appeared on March 2, August 24 and 31, and Sept. 14 and 21, page 533.

**Papers on Madness**—(*continued*).

who regard madness as consisting in unsoundness of mind or in cherishing delusion would scarcely hesitate to certify as mad a man who was trying to cut his own throat or the throats of other people; and although by certifying such a patient the doctor would, and constantly does, abandon in practice his contention that madness consists in unsoundness of mind, he would not perceive the discrepancy between his doctrine and his practice. On the contrary there are alienists who would not hesitate for a moment to sign a certificate for such a patient without pausing for an instant to investigate his mind, and who would yet argue the next moment that madness consists in unsoundness of mind, and in unsoundness of mind alone; such curious inconsistency is there in the minds of those who have to do with mad people.

Even, however, if a patient is undoubtedly mad, as proved by the madness of his acts, it does not follow that he need be certified. There are many mad people whom it is quite unnecessary to certify as mad. If a man has to be restrained in his acts on account of his madness, or if he is placed in the care of anyone who receives payment for boarding, lodging or taking charge of him, then it is necessary that he should be certified, and then to refrain from certifying him is an infringement of the law; but if the conduct of the madman is merely incompetent, so that there is no need for restraint, or if he is treated in his own home, and no one receives payment for taking charge of him, then there is no legal necessity for certification. In fact, many mad people are not actively but passively mad. Their conduct is wanting in certain material respects, but is not, or is but little astray. There are very many elderly people who become demented, or deprived of much of their minds. Their conduct is not actively objectionable, except, perhaps, that they may get into futile, feeble rages, use bad language, resist the efforts of their nurses to dress and undress them, or to feed them, and they must be watched lest they lose themselves in the house or the garden, or stray into the dangers of the streets.

Again, we put off as long as possible, and beyond the time when it is legally obligatory, the certification of young people. An opening blossom is very sensitive to the frost, and the unfolding minds of young people are very apt to be disordered, and their conduct to suffer corresponding disorders, if they are subjected to undue strain at the time when the mind is rapidly expanding, especially if the body is at the same time undergoing rapid development. Under the present system of education, many adolescents of both sexes undergo what is euphemistically called a nervous breakdown, in other words an attack of madness, from working excessively hard and placing too great a strain on body or mind or both, by a too enthusiastic devotion to study, or to both study and athletics, at a time when growth is proceeding rapidly, or is incomplete. The double or triple strain is more than the organisation can bear, and it breaks down and becomes disordered, and the disorder is madness. This is especially apt to occur if any additional strain, such as an attack of bodily illness, is added to the other stresses to which the organisation is subjected; and not even the strongest organisations can withstand the accumulated stress. Readers of Lord Lister's life will remember that even his splendid intellect was disordered from such an accumulation of stresses when he was a student at University College, and probably the majority of young women who take up the medical profession break down in the same way from the same cause. The malady is usually, as in Lister's case, but temporary, and in most cases the patient recovers in a few weeks or months; and in view of the injury that the subsequent career may suffer if the patient is formally certified as insane, it is usual to refrain from certification; and the authorities judiciously wink at this infraction of the law.

